# Septic Cardiomyopathy: Difficult Definition, Challenging Diagnosis, Unclear Treatment

**DOI:** 10.3390/jcm14030986

**Published:** 2025-02-04

**Authors:** George E. Zakynthinos, Grigorios Giamouzis, Andrew Xanthopoulos, Evangelos Oikonomou, Konstantinos Kalogeras, Nikitas Karavidas, Ilias E. Dimeas, Ioannis Gialamas, Maria Ioanna Gounaridi, Gerasimos Siasos, Manolis Vavuranakis, Epaminondas Zakynthinos, Vasiliki Tsolaki

**Affiliations:** 1 3rd Department of Cardiology, “Sotiria” Chest Diseases Hospital, Medical School, National and Kapodistrian University of Athens, 11527 Athens, Greece; gzakynthinos2@gmail.com (G.E.Z.); boikono@gmail.com (E.O.); kalogerask@yahoo.gr (K.K.); jyialamas@gmail.com (I.G.); mar.gounaridi@gmail.com (M.I.G.); ger_sias@hotmail.com (G.S.); vavouranakis@gmail.com (M.V.); 2 Department of Cardiology, University Hospital of Larissa, Faculty of Medicine, University of Thessaly, 41110 Larissa, Greece; grgiamouzis@gmail.com (G.G.); andrewvxanth@gmail.com (A.X.); 3 Critical Care Department, University Hospital of Larissa, Faculty of Medicine, University of Thessaly, Mezourlo, 41335 Larissa, Greece; nikitaskaravidas@gmail.com (N.K.); dimel13@hotmail.com (I.E.D.); vasotsolaki@yahoo.com (V.T.); 4 Cardiovascular Division, Brigham and Women’s Hospital, Harvard Medical School, Boston, MA 02115, USA

**Keywords:** septic cardiomyopathy, sepsis-induced myocardial dysfunction, mitochondrial dysfunction, myocardium-depressing factors, sepsis, myocardial dysfunction, echocardiography, cardiac MRI, troponin, B-type natriuretic peptide, biomarkers, global longitudinal strain, diastolic function, levosimendan, venoarterial extracorporeal membrane oxygenation (VA-ECMO), sepsis phenotypes

## Abstract

Sepsis is a systemic inflammatory response syndrome of suspected or confirmed infectious origin, which frequently culminates in multiorgan failure, including cardiac involvement. Septic cardiomyopathy (SCM) remains a poorly defined clinical entity, lacking a formal or consensus definition and representing a significant knowledge gap in critical care medicine. It is an often-underdiagnosed complication of sepsis. The only widely accepted aspect of its definition is that SCM is a transient myocardial dysfunction occurring in patients with sepsis, which cannot be attributed to ischemia or pre-existing cardiac disease. The pathogenesis of SCM appears to be multifactorial, involving inflammatory cytokines, overproduction of nitric oxide, mitochondrial dysfunction, calcium homeostasis dysregulation, autonomic imbalance, and myocardial edema. Diagnosis primarily relies on echocardiography, with advanced tools such as tissue Doppler imaging (TDI) and global longitudinal strain (GLS) providing greater sensitivity for detecting subclinical dysfunction and guiding therapeutic decisions. Traditional echocardiographic findings, such as left ventricular ejection fraction measured by 2D echocardiography, often reflect systemic vasoplegia rather than intrinsic myocardial dysfunction, complicating accurate diagnosis. Right ventricular (RV) dysfunction, identified as a critical component of SCM in many studies, has multifactorial pathophysiology. Factors including septic cardiomyopathy itself, mechanical ventilation, hypoxemia, and hypercapnia—particularly in cases complicated by acute respiratory distress syndrome (ARDS)—increase RV afterload and exacerbate RV dysfunction. The prognostic value of cardiac biomarkers, such as troponins and natriuretic peptides, remains uncertain, as these markers primarily reflect illness severity rather than being specific to SCM. Treatment focuses on the early recognition of sepsis, hemodynamic optimization, and etiological interventions, as no targeted therapies currently exist. Emerging therapies, such as levosimendan and VA-ECMO, show potential in severe SCM cases, though further validation is needed. The lack of standardized diagnostic criteria, combined with the heterogeneity of sepsis presentations, poses significant challenges to the effective management of SCM. Future research should focus on developing cluster-based classification systems for septic shock patients by integrating biomarkers, echocardiographic findings, and clinical parameters. These advancements could clarify the underlying pathophysiology and enable tailored therapeutic strategies to improve outcomes for SCM patients.

## 1. Introduction

The Third International Consensus Definitions for Sepsis and Septic Shock defines sepsis as a life-threatening organ dysfunction caused by a dysregulated host response to infection [1].

Although septic cardiomyopathy (SCM) has been recognized for over 40 years, it remains incompletely understood [2,3]. Sepsis-induced cardiomyopathy is commonly encountered in the intensive care unit (ICU), with its prevalence among septic patients ranging from 10% to 70% [4]. This wide variation between studies is likely attributable to a lack of formal diagnostic criteria and under-recognition. Additionally, the epidemiologic discrepancies underscore the multifaceted nature of sepsis, including variations in the source and severity of infection, the timeliness of resuscitation efforts, and differences in antimicrobial and hemodynamic treatment strategies.

While findings are varied, some studies suggest that the presence of SCM is associated with a two- to threefold increase in mortality [5,6]. Indeed, the heart is among the many organs affected by sepsis. However, likely due to the absence of a definitive definition, acute heart failure caused by SCM has not been incorporated into widely used severity scoring systems for critically ill patients. This includes systems that rely on data obtained on the first day of ICU admission—such as the Acute Physiology and Chronic Health Evaluation (APACHE), the Simplified Acute Physiology Score (SAPS), and the Mortality Prediction Model (MPM)—as well as those that collect data sequentially over the ICU stay, including the Sepsis-related Organ Failure Assessment (SOFA) and the Multiple Organ Dysfunction Score (MODS).

In the 1980s, Parker and colleagues used radionuclide cineangiography to demonstrate reversible depression in left ventricular ejection fraction (LVEF) and ventricular dilation in a subset of septic patients [2]. These characteristics are now frequently used to describe sepsis-induced myocardial dysfunction [2,7]. With advancements in echocardiography, the term has since expanded to include other cardiac abnormalities, such as diastolic dysfunction, right ventricular (RV) failure, and supranormal LVEF.

Sepsis affects cardiac performance through numerous mechanisms, resulting in considerable heterogeneity in the cardiac responses observed in affected patients. Advances in diagnostic modalities, particularly echocardiography, have facilitated a deeper understanding of these variations. Patient- and disease-specific factors likely contribute to the observed differences in cardiac responses to sepsis. Understanding these factors can help inform a more individualized approach to hemodynamic resuscitation and overall care.

This review aims to present the recent literature focusing on the definition and mechanisms underlying the development of cardiac dysfunction in sepsis. Particular emphasis will be placed on diagnostic approaches, with a focus on echocardiography and its associated findings in sepsis and septic shock. Additionally, the review will explore the potential treatments for sepsis-induced cardiomyopathy.

## 2. Definition

At present, no formal or consensus definition of SCM exists, representing a significant knowledge gap. The complexity of the cardiovascular system, myriad methods of assessment, and variations in the pre-septic state of the heart make it challenging to establish a clear cause-and-effect relationship.

One of the primary challenges in defining SC lies in evaluating cardiac function under the highly variable preload and afterload conditions seen in sepsis. Most review articles and expert opinions agree on several fundamental characteristics of this unique form of cardiac dysfunction. These features include acute systolic dysfunction with reduced contractility of one or both ventricles, or even diastolic dysfunction, in the absence of atherosclerotic coronary artery disease. However, whether isolated diastolic left ventricular (LV) dysfunction or isolated right ventricular (RV) dysfunction should be part of the SCM definition remains a subject of debate [1]. Another notable feature of SC is its rapid reversibility. Recently, Martin et al. [8] proposed that, in addition to reduced ventricular contractility, a combination of LV dilation with normal or low left ventricular filling pressure (LVFP) is a characteristic of SCM. This observation, however, is not new. Earlier studies by Jardin et al. [9] and Bouhemad et al. [10] reported that depressed LV systolic function was associated with normal or low LVFP, unlike the classic pattern of cardiogenic shock, where LV pressures are markedly elevated. These findings suggest that the pulmonary artery catheter, historically used for monitoring, may have underestimated the incidence of LV systolic dysfunction. Jardin et al. [9] reported an average pulmonary capillary wedge pressure (PCWP) of approximately 11 mmHg in patients with reduced LVEF, similar to those with preserved ejection fraction. Similarly, Parker et al. [2] observed an average PCWP of 14 mmHg in patients with LV ejection fraction <45%.

While a dilated left ventricle typically has increased end-diastolic volume and pressure, it seems improbable that well-resuscitated septic cardiomyopathy patients could exhibit LV dilation without a relatively elevated LVFP. Indeed, a meta-analysis reported significantly higher E/e’ ratios (a surrogate for LVFP, measured via tissue Doppler imaging) in non-survivors of sepsis. However, even survivors often exhibited abnormal E/e’ values, indicating a high prevalence of elevated LVFP during sepsis [11].

Interestingly, LVFP in SCM may not reach the levels observed in cardiogenic shock. This phenomenon could be attributed to increased vascular permeability, a hallmark of sepsis, which affects all organs, including the lungs, and therefore pulmonary edema may occur at significantly lower or near-normal LVFP values if colloid oncotic pressure is also reduced, as hypoalbuminemia is frequently present in sepsis.

Parker et al. observed a significant increase in LV compliance and an excessive increase in mean end-systolic and end-diastolic ventricular volumes in patients with severe depression of LVEF (<40%), although most patients with documented septic shock maintained a normal stroke volume. Furthermore, Parker et al. demonstrated a paradoxical relationship between decreased LVEF and lower mortality. Nonsurvivors had normal initial ejection fraction and ventricular volumes that did not change during serial studies [2]. However, this finding was never corroborated in subsequent studies and may have been partly explained by technical errors associated with pulmonary artery catheter measurements. Echocardiographic studies have instead reported only a modest increase in LV size in patients with decreased ejection fraction compared to those with preserved ejection fraction, suggesting a slight increase in LV compliance [12,13].

Echocardiography remains the preferred modality for evaluating SCM due to its widespread availability, noninvasive nature, and repeatability. However, systolic function assessed by LVEF may be overestimated in cases of severe septic vasodilation, potentially limiting its diagnostic and prognostic utility in septic patients [14]. This limitation arises because LVEF does not account for the fluctuating afterload conditions of the left and right ventricles, which also frequently change during sepsis and septic shock.

The lack of a standardized definition of SCM contributes to significant variability in reported incidence rates of LV and/or RV dysfunction, depending on evaluation methods and diagnostic criteria [4]. Furthermore, the timing of echocardiographic evaluation plays a crucial role in this heterogeneity. For example, LV dysfunction was observed in 22% of patients evaluated within 24 h of sepsis onset, increasing to 31.8% when assessed after 72 h [15]. In another study, LV dysfunction was reported in 18% of patients assessed within 6 h of admission, rising to 60% after 72 h [16]. Additionally, studies vary in inclusion criteria; some include patients both with sepsis and septic shock, while others focus exclusively on septic shock requiring high doses of catecholamines, which, by themselves, can influence cardiac function. Additionally, patients in the ICU may or may not be on mechanical ventilation, which also impacts the evaluation of SCM and mainly RV.

Despite the absence of a formalized definition, sepsis-induced myocardial dysfunction is generally characterized as an acute and at least partially reversible condition, not caused by acute coronary syndrome, usually resolving within a few days (usually seven to ten days). It manifests as mono or biventricular systolic and/or diastolic dysfunction with slight LV dilation that is poorly responsive to fluids and catecholamines. However, an LVEF < 40% in a patient with severe sepsis and/or septic shock without coronary syndrome, which improves or reverses in a short period of time, is used most frequently to describe SCM, especially when considering the low vascular resistance that coexists (as explained below).

Finally, although significant progress has been made in understanding SCM, several challenges persist. Its heterogeneity, the absence of standardized diagnostic criteria, and the overlap with other forms of cardiac dysfunction continue to hinder accurate diagnosis and management. Establishing a consensus definition and refining diagnostic approaches will be essential steps toward enhancing the care and outcomes of septic patients.

## 3. Pathophysiology: Mechanisms Proposed for Sepsis-Induced Cardiomyopathy

The mechanisms behind SCM are not fully understood. Initially, SCM was thought to result from global myocardial ischemia due to inadequate coronary blood flow. However, studies have shown preserved or increased coronary blood flow in septic shock patients with myocardial dysfunction, refuting this hypothesis [17]. Further studies showed no significant lactate changes in the coronary sinus, ruling out myocardial ischemia as a cause of LV dysfunction in sepsis [18]. While some studies support coronary ischemia due to microcirculatory hypoperfusion [19], others do not observe coronary microvascular dysfunction [20]. Various mechanisms have been proposed to explain sepsis-associated myocardial changes, arising from pathogen-induced and dysregulated immune responses [19,21].

### 3.1. Myocardium-Depressing Factors

Pathogen-associated molecular patterns (PAMPs) like lipopolysaccharides (LPS) and damage-associated molecular patterns (DAMPs) activate toll-like receptors (TLRs), triggering NF-κB translocation into the nucleus. This results in the transcription of pro-inflammatory cytokines such as IL-1, IL-6, and TNF-α, leading to a “gene storm” [22,23,24,25]. These cytokines are linked to myocardial dysfunction and organ failure in septic shock [26]. In contrast, adaptive immunity and repair mediators like IL-17 and vascular endothelial growth factor (VEGF) are associated with faster sepsis resolution and improved survival [26], although SCM as a distinct group was not studied. An imbalance between these cytokines may promote myocardial dysfunction in some patients, though not consistently observed across studies [27]. Recent studies show that bacterial-infected macrophages release exosomes that exacerbate inflammation, and blocking exosome release in septic mouse models reduced pro-inflammatory cytokines, attenuated cardiac depression, and improved survival [28].

Pilot studies on cytokines in SCM since the 1990s noted transient improvement in ventricular function in septic patients after murine monoclonal anti-TNF antibody administration [29]. However, clinical trials, including the NORASEPT II trial, failed to show survival benefits from anti-TNF monoclonal antibodies [30]. This suggests that TNF-α and IL-1 may play a transient role in early cardiac depression but are not major drivers of persistent myocardial dysfunction in SCM. Instead, excessive nitric oxide (NO) production may perpetuate cardiac depression [23].

### 3.2. Excessive Nitric Oxide Production

Inflammatory cytokines such as IL-1β and TNF-α stimulate inducible nitric oxide synthase (iNOS), leading to excessive nitric oxide (NO) production. Unlike small amounts produced by endothelial (eNOS) and neuronal (nNOS) nitric oxide synthases, sustained NO release can contribute to myocardial depression [31,32]. Excess NO downregulates beta-adrenergic receptors, reducing myocyte responsiveness to adrenergic stimulation [33,34]. Additionally, NO-induced overexpression of cyclic guanosine monophosphate (cGMP) impairs myofilament calcium responsiveness and alters preload and afterload [35]. Excessive NO also worsens mitochondrial dysfunction by increasing permeability, as seen in septic mouse models [36,37].

### 3.3. Mitochondrial Dysfunction

Mitochondrial function is vital for ATP production in energy-demanding organs like the heart. During sepsis, dysfunction arises from morphological changes, oxidative damage to mitochondrial DNA by PAMPs [38], and increased permeability from NOS overexpression [37]. Downregulation of mitochondrial protein synthesis genes during early inflammation [39] further impairs ATP production. Hearts from septic patients show reduced expression of mitochondrial ATP-production-related genes [40]. Oxidative stress worsens myocardial dysfunction by disrupting calcium homeostasis and mitochondrial efficiency [41]. Drugs used in critical care can also inhibit mitochondrial function [42].

Hyperlactatemia in sepsis results not only from tissue hypoxia but also from adrenergic stimulation increasing myocardial energy demand [43]. Non-mitochondrial ATP production is unsustainable and a short-term compensatory mechanism, leading cells into a “hibernation” state. In the human heart, hibernation is a phenomenon where cardiomyocytes decrease activity in response to reduced myocardial perfusion, potentially explaining both depressed cardiac function and its reversibility in SCM [44].

### 3.4. Calcium Dysregulation

Inflammatory cytokines disrupt calcium handling in cardiomyocytes. TNF-α-induced methylation of the sarcoplasmic reticulum (SR) calcium ATPase (SERCA) promoter suppresses calcium reuptake [45]. In septic murine models, preventing SERCA suppression improved cardiac function [46]. Impaired calcium reuptake reduces diastolic relaxation, while reduced calcium release affects systolic contraction, contributing to SCM pathophysiology.

### 3.5. Adrenergic Overstimulation

The hyperdynamic heart with low systemic vascular resistance (SVR) is the typical cardiac response to sepsis. It compensates for vasoplegia or inadequate intravascular volume due to a hyperadrenergic state-through activation of beta-1 receptors-from excessive endogenous and/or exogenous catecholamine stimulation. Sympathetic hyperactivation can lead to myocardial dysfunction via tachycardia, shortened diastole, and LV filling, or by converting adrenergic G-protein coupling from a stimulatory to an inhibitory response [19]. Stress-induced cardiac lesions from adrenergic stimulation may also contribute to SCM [47]. Research shows that hyperdynamic LV function is linked to increased mortality [48,49]. Ιn a recent study, patients with supranormal LVEF had worse outcomes compared to those with normal or low LVEF [48]. This group had lower SVR, potentially indicating persistent vasoplegia as the driver of mortality. These patients also had higher heart rates, so the hyperdynamic state from excessive catecholamine release may also contributed to a latent SCM. This subset of patients with hyperdynamic state might explain why lower EF patients fared better in earlier studies.

Beta-blockers have been studied to reduce heart rate and improve stroke volume in septic patients with tachycardia. A meta-analysis found that using short-acting beta-blockers like esmolol or landiolol after resuscitation led to decreased mortality [50].

This may also be related to the fact that continuous adrenergic stimulation causes a downregulation of beta receptors, reducing their density on the myocyte walls. This could explain the refractory response to catecholamines observed in some patients with septic shock [19].

Therefore, beta-blockers may also help achieve de-catecholaminization, known to improve cardiac function in heart failure patients. Similarly, dexmedetomidine (DEX), a sedative α-2 adrenoreceptor agonist, has shown benefits in reducing heart rate and sinus node activity due to its sympatholytic effects [51]. With presynaptic alpha2-adrenoreceptor inhibiting sympathetic release of catecholamines, DEX potentially could offer decatecholaminization [52].

In a recent study DEX significantly reduced HR, persistent atrial fibrillation, and markers of inflammation, such as C-reactive protein. However, what stood out new in this study was that DEX was used to control HR for those patients with septic shock and tachycardia for decatecholaminization [53].

While these studies do not specifically target hyperdynamic LVEF, beta-blockers, dexmedetomidine, and other agents reducing catecholamine overstimulation may offer potential therapy, though further research is needed.

### 3.6. Myocardial Edema

Recent studies highlight myocardial edema (ME) in SCM pathogenesis [54]. Increased microvascular permeability and reduced lymphatic clearance lead to fluid accumulation in the myocardial interstitium. Cytokines like TNF-α, IL-1β, and IL-6 increase endothelial permeability, worsening ME [55]. Lymphatic drainage is further impaired by reduced myocardial contractions in sepsis [56], as heart lymphatic vessels rely on contractions for fluid propulsion [57]. Even a 3.5% increase in myocardial water content can reduce cardiac output by 40% due to increased ventricular stiffness [58,59,60,61]. Moreover, cardiac function does not immediately improve upon the resolution of ME.

In summary, the pathophysiology of SCM involves a combination of macro- and microcirculatory changes, adrenergic receptor downregulation, impaired calcium cycling, inflammation-induced myocardial depression, mitochondrial dysfunction, and myocardial edema. Cytokines and nitric oxide appear to play central roles in driving these processes. Addressing these mechanisms could inform future therapeutic strategies for managing septic myocardial dysfunction.

## 4. Diagnosis

Clinicians should consider a diagnosis of SCM in all septic patients with sepsis-associated organ dysfunction, particularly in cases of septic shock requiring vasopressor therapy [4,19,21]. Echocardiography is the most useful technique for bedside evaluation. While intrinsic myocardial contractility can be accurately measured through pressure-volume loop analysis using a conductance catheter [62], multicrystal sonomicrometry [63], or radionuclide techniques [64], these methods are not feasible for bedside use, especially in critically ill patients.

### 4.1. Echocardiography

Echocardiography is the primary imaging modality for assessing cardiac function in septic patients. In fact, it is the most useful technique for evaluating SCM. It provides a dynamic visualization of the heart, enabling the measurement of wall motion, EF, and ventricular filling. The method is simple, accessible, cost-effective, repeatable, and can be performed bedside in critically ill patients.

Transthoracic echocardiography (TTE) is typically the first choice due to its non-invasive nature. However, in patients with limited image quality—due to obesity, pulmonary factors (e.g., chronic obstructive pulmonary disease, mechanical ventilation with increased PEEP)—transesophageal echocardiography (TEE) offers clearer images.

Conventional echocardiography can evaluate:

- The structural integrity of the heart, including valve pathologies and assessing chambers size and wall thickness.
- Left and right Ventricular function, in terms of systolic and diastolic performance. However, certain limitations should be considered, especially in patients with sepsis or septic shock and potential SCM.
- The existence of pericardial disease.

The wide variation in reported incidence of SCM [4,14], likely reflects differing definitions, echocardiographic techniques, timing of assessment during the clinical course, and the dynamic nature of cardiac functional changes. Factors such as fluid resuscitation, inotrope and vasopressor therapy, and mechanical ventilation can influence venous return, cardiac contractility, pulmonary vascular resistance, and left ventricular afterload, thereby affecting traditionally measured echocardiographic parameters.

### 4.2. Echocardiography of the Left Ventricle

#### Systolic Function

The illusory normalization of LVEF in cases of reduced afterload in distributive shock presents a diagnostic challenge. LVEF is not a sensitive indicator of myocardial contractility, as it reflects the relationship between LV myocardial contractility and afterload.

To understand this, the term “contractility” must be clarified: it refers to the intrinsic property of the myocardial fiber, independent of preload and afterload. Consequently, during septic shock, LVEF ceases to reflect LV contractility accurately and instead mirrors to some extent the degree of reduced systemic SVR, which corresponds to the level of peripheral vascular paralysis.

As Robotham et al. [65] demonstrated, the same LVEF value may correspond to vastly different levels of intrinsic LV contractility. For example, an EF of 60% could indicate severe impairment of LV contractility if afterload is markedly reduced. In a compelling case, a patient with septic shock showed an EF of 70% after initial resuscitation, which dropped precipitously to 40% after a few hours of norepinephrine infusion. This change unmasked the impaired contractility when normal LV afterload was increased [66].

Given that severe reductions in afterload can obscure cardiac impairment, either sophisticated echocardiographic techniques or global heart function parameters that account for afterload dependency are required. One such parameter, afterload-related cardiac performance (ACP), calculates the percentage of cardiac output (CO) in a septic patient relative to the CO of a healthy heart under equivalent SVR reduction. The calculation of ACP show that approximately 50% of septic patients suffer from impaired cardiac function, and mortality increases with severity [14].

Similarly, it has been hypothesized [67] that afterload-adjusted LVEF and LV outflow tract velocity-time integral (VTI)—a surrogate for stroke volume—could provide insights into stratifying SCM severity. In mechanically ventilated septic shock patients, simultaneous echocardiographic and PiCCO examinations demonstrated that LVEF and VTI were inversely correlated with SVR. Afterload-adjusted LVEF, defined as the measured-to-predicted LVEF ratio, stratified SCM severity into mild (≤90%), moderate (80–89%), and severe (<80%) dysfunction.

**Figure 1:**
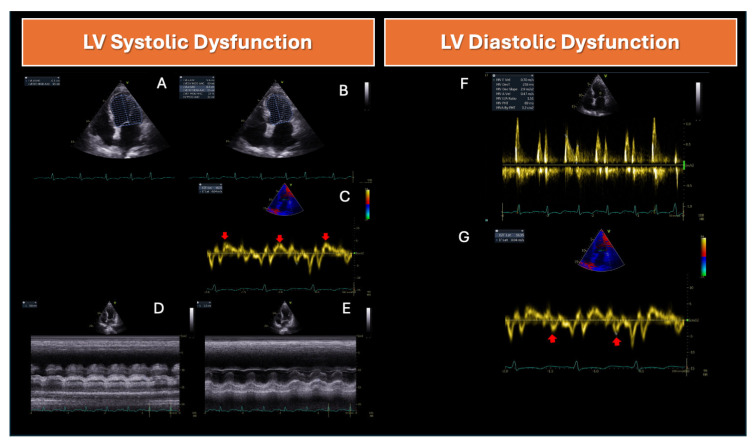
Echocardiographic markers of left ventricular systolic and diastolic function. (**A**,**B**): Left ventricular ejection fraction (EF) estimation through the Simpson’s method in a patients with SCM. LVEF was 27%. (**C**): Tissue Doppler imaging evaluating the systolic velocity of the lateral mitral annulus (S’) which was very low in the present patient (0.04 m/s) (red arrows). (**D**,**E**): Mitral Annular Plane Systolic Excursion (MAPSE) at the septal and lateral mitral annulus which was 0.8 and 1.3 mm, respectively, indicating severe LV systolic dysfunction. (**F**,**G**): transmitral flow and tissue Doppler imaging at the mitral annulus to evaluate diastolic LV function. E/e’ was 16.35, severely increased, indicating diastolic dysfunction in a patient with urinary sepsis and previously normal cardiac function. E’ was 0.04 cm/s also indicating severe diastolic dysfunction.

Initially, SCM diagnosis was based solely on depressed LVEF (<45%) [2]. Systolic function is commonly assessed using LVEF measured by Simpson’s method of disks or even by subjective “eyeball” estimates from experienced clinicians (Figure 1). Fractional area contraction at the LV midpoint is another method [68,69], but as previously discussed, LVEF is an imperfect indicator of intrinsic myocardial contractility.

A retrospective analysis in an intensive care unit, examining echocardiographic findings within three days of admission, found that most patients had LVEF between 55% and 70%. However, the highest in-hospital mortality occurred in patients with LVEF < 25% and those with hyperdynamic myocardium (LVEF > 70%). Patients with LVEF < 25% primarily died from cardiogenic shock, whereas those with hyperdynamic myocardium often died from severe septic shock, where EF reflected a hyperadrenergic state driven by excessive endogenous and exogenous catecholamine stimulation [70]. Therefore, in critically ill patients, LVEF should be reported alongside inotropic and vasopressor support levels and the degree of shock. In another study, LVEF was used to categorize septic systolic ventricular dysfunction severity: mildly abnormal (LVEF 41–51%), moderately abnormal (30–40%), or severely abnormal (<30%). However, preload and afterload conditions, which vary constantly in sepsis, were not considered in this study. This failure to account for dynamic conditions may explain inconsistencies across studies in association with mortality [71].

Therefore, rather older studies but also a recent one reported that decreased LVEF was associated with higher mortality [72,73], while others found no survival differences based on LVEF [74,75]. Older studies even reported improved survival [76].

Nonetheless, echocardiographic variables hold both diagnostic and prognostic value in SCM. Advanced echocardiographic techniques—such as tissue Doppler imaging (TDI), myocardial strain analysis using speckle-tracking echocardiography (STE), and three-dimensional echocardiography—offer greater detail regarding myocardial function and hemodynamics [71]. However, three-dimensional echocardiography alone remains an imperfect measure of LV systolic function, as it is influenced by LV contractility, preload, and mainly afterload conditions.

### 4.3. Tissue Doppler Imaging (TDI)

Peak systolic velocity measured at the mitral annulus (as determined by the S’ value using TDI) is another appropriate surrogate for estimating LV systolic function (Figure 1). It is considered less dependent on loading conditions compared to LVEF [77,78]. Studies have shown that survivors of sepsis have lower mitral S’ values than non-survivors, with a 90-day mortality predictive cut-off at 9 cm/s [79]. However, the correlation between S’ and cardiac output (CO) was stronger in heart failure (HF) no SCM patients compared to septic patients (R = 0.48 vs. R = 0.34, respectively). This correlation was statistically significant only in HF patients, suggesting that individual S’ measurements may not reliably estimate CO in sepsis [80]. Low SVR in sepsis reduces afterload, potentially increasing mitral S’ [80]. This is also supported by the observation that mitral S’ correlates with LVEF that overestimates contractility [79,81], although, mitral S’ has been found to predict disease severity in septic patients newly admitted to emergency units [81].

However, a meta-analysis investigating patients with sepsis found no differences in S’ values between survivors and non-survivors [82]. Finally, it must be emphasized that the measurement of S’ is limited by angle dependency. It measures unidirectional peak systolic velocity in a single segment. Therefore, both sides of the mitral annulus—the free wall and septal edges—must be evaluated from a four-chamber view.

### 4.4. Mitral Annular Plane Systolic Excursion (MAPSE)

Mitral annular plane systolic excursion (MAPSE) also plays an important role in assessing LV function [83,84]. However, data regarding its use in different clinical contexts remain limited, given the diversity of cardiovascular pathologies [85]. Brault et al. [86] recently demonstrated a positive correlation between septal MAPSE ≤ 1.2 cm and LV systolic dysfunction. Conversely, a septal MAPSE ≥ 1.2 cm predicted normal LVEF in 94% of cases. The authors concluded that septal MAPSE is an easily measurable parameter at the bedside and may help clinicians detect early LV systolic dysfunction, particularly in situations where myocardial strain measurements are not feasible (Figure 1).

### 4.5. Global Longitudinal Strain (GLS)

Strain imaging represents a relatively modern echocardiographic approach to assess myocardial function over time. Global longitudinal strain (GLS), calculated using speckle-tracking echocardiography (STE), is the most commonly used parameter [87].

**Figure 2:**
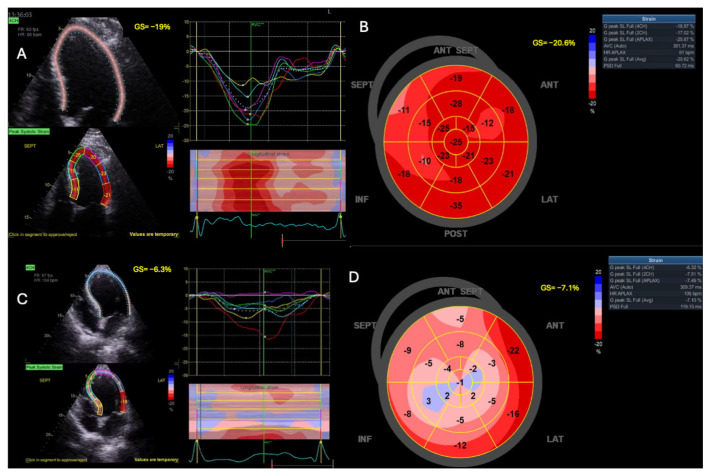
(**A**). Speckle-tracking analysis of a patient with normal systolic left ventricular (LV) function. Two-dimensional image showing speckles within the LV being tracked by the ultrasound Machine Software (EchoPAC Software version 203). Graphical representation of the movement of speckles throughout the cardiac cycle (*x*-axis, longitudinal strain; *y*-axis, time in msec), with each line representing a different segment of the LV; large negative values represent movement of speckles towards one another during contraction representing normal function. (**B**). Bullseye map showing global longitudinal strain values throughout the LV. (**C**). Speckle-tracking analysis of a patient with sepsis and severely reduced left ventricular (LV) systolic function. A 2D image showing speckles within the LV being tracked by the ultrasound machine software. Graphical representation of movement of speckles throughout the cardiac cycle (x-axis, longitudinal strain; y-axis, time in msec) with each line representing a different segment of the LV; note smaller negative values with variable time to peak strain representing reduced LV function with mechanical dyssynchrony. Bullseye map showing global longitudinal strain values throughout the LV; blue zones represent areas of the LV where there is lengthening of the segments during systole rather than shortening (**D**).

**Figure 3:**
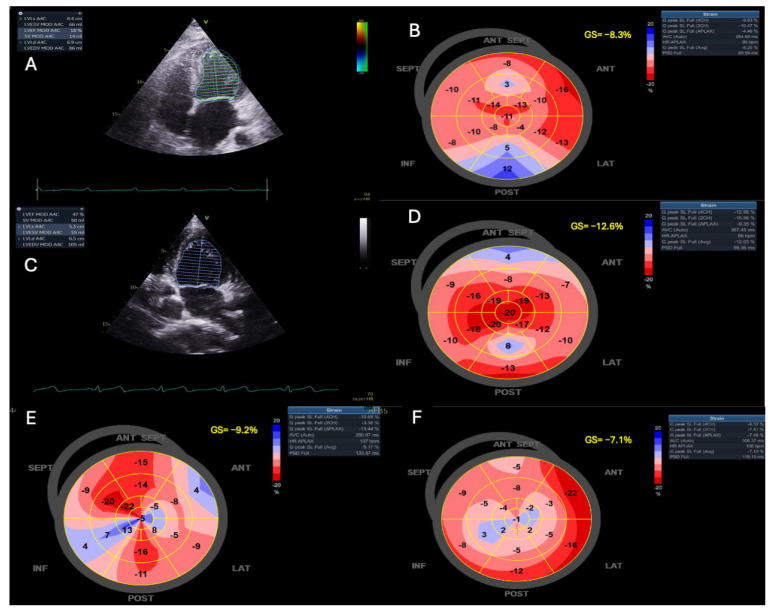
Speckle-tracking analysis in patients with SCM. (**A**). A patient with severely decreased LV EF (18%) measured with the Simpson’s method and the (**B**). corresponding GLS with STE which was also found severely decreased. (**C**). A patient with septic shock and mildly reduced LVEF (47%). The corresponding GLS (**D**) was found severely decreased, better depicting the depressed cardiac contractility which was probably masked due to the decreased peripheral vascular resistances. (**E**,**F**) present GLS examinations in patients with SCM. Interesting is the difference in the distribution of the affected myocardial regions presenting altered contractility.

STE uses a semi-automated computer algorithm to track myocardial regions (speckles) during the cardiac cycle. During systole, as fibers contract, the speckles move closer together, resulting in negative values. Larger negative values indicate greater deformation and improved LV function. Normal GLS values typically range around −20% [88] (Figure 2 and Figure 3).

GLS is a reliable surrogate for LV contractility and is less dependent on angle and loading conditions compared to other methods for evaluating systolic dysfunction [89,90]. Additionally, GLS enables early detection of subclinical myocardial dysfunction during illness [90,91]. Thus, assessing GLS is particularly valuable for detecting early myocardial damage during sepsis (Figure 3). However, its use in ICUs is often limited by technical challenges, including the need for good endocardial visualization and high frame rates [15,71]. Despite these limitations, GLS has been recognized as the most reproducible measure of cardiac function [71].

In patients with septic shock, GLS detected changes in myocardial contractility in 70% of cases, compared to only 32% detected using LVEF [15]. In this context, GLS measured on day 1 predicted reduced LVEF on subsequent days [15].

Lower (less negative) GLS values are also associated with higher mortality in sepsis, whereas LVEF lacks such a correlation [92]. GLS can detect early cardiac dysfunction even in patients with preserved LV EF (>50%). A GLS value ≥ −13% was found to independently predict ICU and hospital mortality, outperforming LVEF in differentiating survivors from non-survivors. Moreover, GLS demonstrated superior prognostic value compared to the APACHE II score, aiding in early identification of high-risk septic patients [93].

Dalla et al. [94] observed that 50% of septic patients with LVEF > 50% exhibited impaired LV function (GLS > −15%), compared to only 8.7% of non-septic patients. This highlights the ability of STE to detect systolic dysfunction before conventional imaging, including EF and CO, reveals abnormalities.

Ng et al. [95] explored differences in myocardial dysfunction severity between sepsis and septic shock, showing that GLS detected significantly more impairment in patients with septic shock (−14.5% vs. −18.3%). This difference was not evident using LVEF. Moreover, GLS demonstrated reversibility during recovery, albeit with modest changes (−14.5% vs. −16.0%, *p* = 0.010). Contradicting this, De Geer et al. [96] observed that LV GLS remained unchanged over time. This suggests that decreased GLS may represent subtle myocardial changes in septic shock that persist even after clinical recovery.

A recent systematic review and meta-analysis evaluated mortality in patients with septic cardiomyopathy identified by GLS. The study, which included 1.678 patients from 14 studies, reported a survival rate of 69.6%. A more negative GLS was significantly associated with better survival, with a mean difference (MD) of −1.45% (*p* < 0.0001). In a secondary analysis, higher LVEF values were also associated with improved survival, although the association was weaker compared to GLS (MD = 2.44%, *p* = 0.02). These findings confirm the prognostic value of GLS in septic patients [97].

An earlier meta-analysis of 794 patients similarly found that less negative GLS values correlated with higher mortality in severe sepsis or septic shock. However, in this analysis, no significant association was observed between LVEF and mortality within the same patient population [92].

In a recent study involving 176 septic patients, 96.6% were receiving norepinephrine, and 10.4% were also receiving argipressin. Despite a nearly normal mean EF of 57.8 ± 1.1%, significant LV systolic dysfunction (LVEF < 40%) was observed in 21 patients (11.9%), with severely decreased EF (<30%) in 7 patients (4%). However, GLS was markedly reduced (−13.3 ± 0.3%). Among these patients, 87.5% exhibited GLS > −18%, while 56.5% had GLS > −15.9%, and 46.2% had GLS > −14% [98]. This underscores the sensitivity of GLS in identifying myocardial impairment that may not be detected using LVEF in septic patients with normal or near-normal EF.

#### Diastolic Function

Diastole is the primary determinant of LV compliance in the absence of infiltrative or restrictive cardiac diseases [99]. Left ventricular end-diastolic pressure (LVEDP) tends to increase as diastolic function deteriorates. This phenomenon can occur in septic shock, particularly after administering large fluid volumes, despite the fact that LVEDP may initially be low in SCM. Left ventricular diastolic dysfunction (DD) is frequently observed in severe sepsis and septic shock (Figure 1) [100].

The adverse effects of DD in SCM are significant. One potential explanation is that elevated LVEDP increases pressure in the pulmonary circulation, the right heart, and peripheral tissues. This results in higher extravascular lung water (EVLW) and tissue edema. Indeed, LVEDP does not need to rise significantly for EVLW to increase in the lungs, especially when sepsis is accompanied by increased vascular permeability and acute respiratory distress syndrome (ARDS), ultimately hindering successful weaning from mechanical ventilation [101].

The standard method for evaluating diastolic function is TDI, which measures the velocity of the mitral annulus during early diastole (e’ wave). The e’ wave reflects LV relaxation, with lower values indicating worse diastolic function [99]. The e’ wave is considered one of the most load-independent parameters for assessing intrinsic DD [102]. The ratio of peak early transmitral inflow velocity (E) (measured via pulsed-wave Doppler) to the e’ wave (E/e’) correlates with LVEDP. A higher E/e’ ratio suggests elevated LVEDP and low LV compliance [102]. However, the accuracy of the E/e’ ratio as a surrogate for LVEDP is less robust in septic cardiomyopathy compared to congestive heart failure. Consequently, E/e’ should not be regarded as a reliable indicator of LVEDP in sepsis and septic shock [103].

Thresholds for defining LV diastolic dysfunction in the ICU include TDI-derived e’ velocity values of <10 cm/s at the lateral annulus and <7 cm/s at the septal annulus, as well as E/e’ ratios > 13 at the lateral annulus and >15 at the septal annulus. These parameters are considered relatively load-independent and are therefore more applicable in critically ill patients [104].

The prognostic significance of e’ and E/e’ in septic patients has been extensively studied, yielding mixed results. In a study by Landesberg et al., a mitral e’ < 8 cm/s emerged as the strongest predictor of mortality in septic-induced myocardial dysfunction (OR = 0.7, *p* < 0.001). This association remained significant even after adjusting for the APACHE II score, low urine output, low CI, and hypoxemia [72]. The heightened mortality risk in patients with DD may stem from their limited ability to tolerate fluids [105].

Similarly, two other studies demonstrated that non-survivors had lower e’ values compared to survivors [72,106,107]. Additionally, elevated E/e’ was found to independently predict in-hospital mortality in two other investigations [107,108].

A meta-analysis by Sanfilippo et al. [109]. reported a prevalence of DD (using septal e’) of 48% and a relative risk of death of 1.82 (95% CI: 1.12–2.97, *p* = 0.002). However, the included studies lacked consistent cutoff values. A subsequent meta-analysis showed that both e’ and higher E/e’ values were strongly associated with mortality in septic patients. Notably, e’ measured at the lateral annulus had a stronger prognostic association than e’ measured at the septal annulus [11].

A separate meta-analysis by the same authors, focusing on mechanically ventilated patients, revealed that difficulty in weaning from mechanical ventilation was associated with worse diastolic function and increased LV filling pressure [101]. Similarly, a previous study demonstrated that LV diastolic dysfunction significantly impacts weaning outcomes in critically ill patients with preserved LV systolic function. Specifically, a lateral mitral E/e’ ratio > 7.8 prior to a spontaneous breathing trial (SBT) identified patients at high risk for weaning failure. Notably, lateral E/e’ measured before the SBT was the only independent predictor of weaning failure [110].

### 4.6. Echocardiography of the Right Ventricle

#### 4.6.1. Pathophysiology and Contributing Factors

The pathophysiology of RV dysfunction in sepsis is multifactorial. Patients in septic shock, typically treated in ICUs and requiring mechanical ventilation, may develop or worsen RV dysfunction due to several factors. Septic cardiomyopathy itself, as well as mechanical ventilation-associated hypoxemia and hypercapnia, particularly in cases complicated by acute respiratory distress syndrome (ARDS), can increase RV afterload and exacerbate RV dysfunction [111]—even with protective ventilation strategies [112].

Since 2007, Jardin et al. [113] highlighted the necessity of optimizing ventilator settings to avoid RV dysfunction, which may progress to acute cor pulmonale (ACP). As they reported in their editorial “Is there a safe plateau pressure in ARDS? The right heart only knows”, RV dysfunction severity and ACP incidence are strongly correlated with increased mortality; increasing ventilator plateau pressure exacerbates ACP occurrence.

#### 4.6.2. Mechanical Ventilation and PEEP Considerations

Not only plateau pressure but also relatively high positive end-expiratory pressure (PEEP) can worsen RV dysfunction. In a recent study [114], a 30% reduction in PEEP from initial levels significantly improved RV function, even when the initial PEEP was lower than what the Surviving Sepsis Campaign guidelines suggest based on FiO_2_ requirements [115]. This reduction led to notable changes, including a significant decrease in RV end-diastolic volume, improved RVEF, and better RV longitudinal strain. Moreover, PVR decreased significantly and RV coupling was improved [116].

These findings underscore the importance of evaluating RV myocardial performance and PVRs when determining whether RV dysfunction in sepsis stems from septic cardiomyopathy or mechanical ventilation [116].

Even in patients with lungs exhibiting normal compliance (CRS) and protective ventilation (tidal volume less than 6 mL/kg), a high PEEP can cause hyperinflation and RV dysfunction [117]. Overdistension, marked by worsening PaCO_2_ and CRS, indicates low potential for recruitment [118], leading to increased PVRs and possible RV dysfunction. However, recruitment maneuvers and/or increased PEEP may reopen collapsed alveoli, ultimately decreasing afterload and facilitating RV ejection [119]. Consequently, mechanical ventilation settings, including PEEP and tidal volume, must be carefully adjusted in cases of suspected RV dysfunction in SCM. Moreover, pulmonary hypertension—affecting the RV—is observed when LVEDP is elevated. Thus, assessing LV diastolic function via e’ and E/e’ ratios is crucial before attributing RV failure severity exclusively to SCM.

##### Assessment of RV Dysfunction via Echocardiography

Right ventricular (RV) dysfunction may be based on ASE [88,120,121] RV function can be affected by several factors, as already mentioned. A combination of measurements can better evaluate RV dilation and dysfunction. Therefore, the ratio of the right ventricular end-diastolic area (RVEDA) to the left ventricular end-diastolic area (LVEDA) (RVEDA/LVEDA) is used for RV dilation (>0.6). When available, 3D echocardiography should be used to estimate RV volumes and RV ejection fraction (RVEF). RV contractility, in addition to measuring RV Fractional Area Change (RVFAC), the Tricuspid Annular Plane Systolic Excursion (TAPSE), the systolic velocity of the annulus of the tricuspid valve (RV S’), and two-dimensional speckle-tracking echocardiography (2D-STE), to assess longitudinal systolic strain (RV-LS) may be used [88,122,123]. In a possible SCM and especially in mechanical ventilation, at least two of the following indices are important to be present: RVFAC ≤ 35%, RV s’) ≤ 10 cm/s, TAPSE ≤ 16 mm, RV-LS) > −20%, RVEF < 44% (assessed via 3D echocardiography) [120].

Pulmonary afterload must be estimated initially by right Pulmonary Arterial Systolic Pressure (PASP) using the simplified Bernoulli equation. However, PVR is indirectly estimated by combining the PASP (calculated via Tricuspid Regurgitation velocity) with the ratio of PASP to RVOT velocity-time integral (PASP/VTIRVOT). This ratio better reflects changes in PVR by integrating PASP and cardiac output [124,125,126]. Additional information about increased PVR could be inferred from: Right Ventriculo-Arterial Coupling (VACR) using the TAPSE/PASP ratio [127,128].

#### 4.6.3. Challenges in Echocardiographic Diagnosis of RV Dysfunction

Given the multifactorial limitations outlined above regarding the causes of RV dysfunction (apart from SCM), echocardiographic diagnosis is both challenging and highly dependent on the experience of the examiner, as well as the diagnostic technique used. The RV’s complex shape makes geometric evaluation difficult, and indices such as TAPSE and RV s’ primarily assess longitudinal function, limiting their ability to provide a comprehensive picture of RV performance. Moreover, the ASE criteria for assessing RV function are not universally adopted by all researchers, leading to variability in diagnostic practices.

As a consequence, the reported prevalence of RV dysfunction in sepsis varies widely (31–83%) [74,108] depending on parameters used and study methodologies. It is generally observed in about 50% of patients with severe sepsis or septic shock [11,129].

In a retrospective cohort study conducted by Vallabhajosyula et al. [130] with 388 ICU septic patients, where 55% of the patients were ventilated, 55.2% of the patients had both RV and LV dysfunction, and 25.8% had isolated RV dysfunction (defined by TAPSE < 16 mm and tricuspid s’ < 0.15 cm/s). It was shown that only isolated RV dysfunction was an independent predictor of one-year survival (HR = 1.6), whereas combined RV/LV dysfunction did not show such a relationship.

A relatively older meta-analysis of 412 septic and septic shock patients found no association between RV function and mortality [131].

TAPSE is likely the easiest and most reproducible measure of RV function [88]. In a cohort study of 120 critically ill patients, not including septic patients, an arbitrary TAPSE cutoff of 2.4 cm was used. The study found that TAPSE < 2.4 cm was the best predictor for in-hospital and long-term mortality [88].

In another study, RV dysfunction was reported in 42% of 282 ventilated patients in septic shock. In this study, RV failure was defined as a combination of RV dilation (RV/LV end-diastolic area > 0.6) and CVP ≥ 8 mmHg [111]. As these researchers had also measured other parameters defining RV dysfunction, it was found that 63.5% of patients with RV failure had a normal TAPSE [111].

Right ventricular strain using STE may be a promising tool to detect RV dysfunction, but the associations between RV strain and TAPSE or other parameters used to assess RV function are moderate. Further studies are needed to determine whether RV dysfunction is a consequence of abnormal loading conditions or intrinsic myocardial dysfunction [11].

Orde et al. [132] reported that reduced RV free wall strain (by STE) was associated with 6-month mortality (−16.0 ± 5.7% vs. −19.3 ± 4.9%, *p* ≤ 0.05). Lanspa et al. [133] measured echocardiographic parameters on 393 critically ill patients with severe sepsis or septic shock within the first 24 h of ICU admission. They defined RV dysfunction as FAC < 35% or TAPSE < 1.6 cm, and LV systolic dysfunction as LVEF < 45% or longitudinal strain > −19%. RV and LV dysfunction were common (48% and 63%, respectively). Patients with RV dysfunction had higher 28-day mortality (31% vs. 16%, *p* = 0.001). In a multivariable regression model, RV dysfunction was also associated with increased mortality. In this study, RV strain was also measured. The correlation between RV strain, TAPSE, and FAC was examined, and the authors found modest associations between RV strain and TAPSE and RV strain and FAC. Additionally, in the multivariable regression analysis performed among strain and clinically relevant covariates, no association between RV strain and mortality after adjustment was shown. However, RV free wall strain was worse in patients with RV dysfunction (−15.9% vs. −19.7%; *p* < 0.001). Therefore, more studies are needed to determine whether traditional parameters or RV strain is better for assessing RV dysfunction.

**Figure 4:**
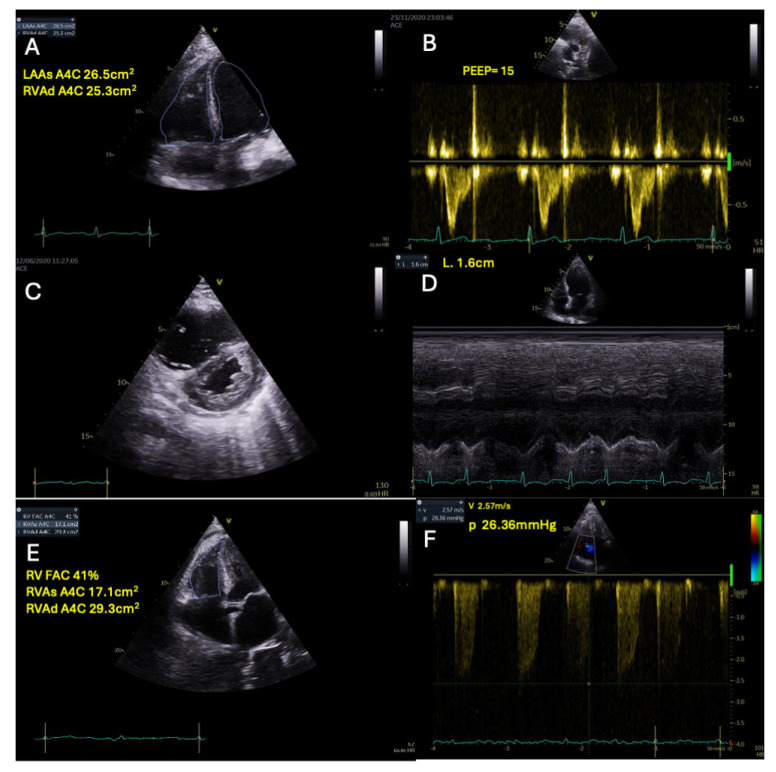
Indices of RV function. (**A**). Assessment of RV size through the evaluation of RV end diastolic area) (RVEDA) to LVEDA. (**B**) RV outflow tract velocity Time Integral (RVOT VTI). The ascending part of the RVOT VTI envelop presents a midsystolic notch which is indicative of increased pulmonary vascular resistances (PVRs). (**C**). D shape of the LV at the parasternal short axis indicating Acute Core Pulmonale (ACP) due to increased RV pressures. In order to conclude that ACP is due to sepsis (SCM) it is essential to exclude all other features that result to RV dusfunction. (**D**). Tricuspid annular Plane Systolic Excursion (TAPSE) which is borderline. (**E**). Fractional Area Change quantification through the equation [RVED area − RVESA)/RVESA]. (**F**). Estimation of RV Systolic pressure through the velocity of the envelope of tricuspid regurgitation. Using the Bernouli equation. Using the above measurements, TAPSE/PASP, a derived variable indicating right ventriculoarterial; coupling, can be evaluated. Moreover, PASP/VTI RVOT can indicate the value of PVRs.

Historically, as a Swan-Ganz catheter is not usually used anymore, Vincent et al. [134] measured RVEF by the thermodilution technique in a series of 127 consecutive critically ill patients monitored with a modified pulmonary artery catheter equipped with a fast-response thermistor. Thermodilution RVEF was significantly lower in septic shock (23.8 ± 8.2%, 93 measurements) than in sepsis without shock (30.3 ± 10.1%) or in the absence of sepsis or cardiopulmonary impairment (32.5 ± 7.1%). RVEF decreased from 35.1 ± 9.8% to 24.2 ± 10.4% (*p* < 0.01) during the development of septic shock and increased from 25.0 ± 7.6% to 29.8 ± 8.5% (*p* < 0.05) during recovery in 14 patients. Initial RVEF in septic shock was 27.8 ± 8.6% in the 11 patients who survived, but only 20.9 ± 6.7% (*p* < 0.02) in the 23 patients who eventually died (Figure 4).

### 4.7. Cardiac MRI

MRI is a promising technique in the evaluation of SCM. The primary limitations for the widespread use of cardiac MRI in clinical practice are the duration of the examination, particularly when dealing with hemodynamically unstable, mechanically ventilated patients. However, cardiac MRI has been reported as a safe and feasible method for critically ill, sedated, and ventilated patients, with breath holds being necessary for the reliability of the MRI, achieved through manual ventilation breaks. These procedures are typically conducted using extensive monitoring and experienced staff, as demonstrated in a recent study [135].

A 2014 MRI study performed on two patients with SCM showed LVEF of 24% and 40%, respectively, and a mildly to moderately increased size of left ventricular end-diastolic volume (LV EDV) (58 and 88 mL/m^2^, respectively). However, homogeneous myocardial enhancement was observed in T2-weighted images, compatible with myocardial edema, and viability was confirmed in all analyzed myocardial segments, with no evidence of myocarditis. Additionally, no late gadolinium enhancement was detected in the LV wall, suggesting the presence of viable myocardium. Remarkably, within a few days, myocardial dysfunction and dilatation were completely reversed in both patients, and myocardial perfusion and viability were normal, with no signs of scar tissue or inflammation. Six weeks later, an MRI revealed a complete recovery of cardiac contractility in one patient [136].

Muehlberg et al. [135], studied twelve patients with septic shock who underwent cardiac MRI within 48 h after the initial peak of catecholamines, alongside transthoracic echocardiography at 48 and 96 h after the MRI. Left ventricular ejection fraction was assessed using both imaging techniques. Patients received gadoteridol for extracellular volume quantification and late gadolinium enhancement imaging. Nine patients had impaired systolic function (LVEF < 50%; mean 39.8 ± 5.7%), while three had preserved LVEF (mean 66.9 ± 6.7%). Global longitudinal strain was impaired in both subgroups (LVEF impaired: 11.0 ± 1.8%; LVEF preserved: 16.0 ± 5.8%; *p* = 0.1). All three patients with initially preserved LVEF died during their hospital stay, while in-hospital mortality in patients with initially impaired LVEF was 11%. Upon echocardiographic follow-up, LVEF improved in all previously impaired patients at 48 h (52.3 ± 9.0%, *p* = 0.06) and 96 h (54.9 ± 7.0%, *p* = 0.02). Patients with impaired systolic function had elevated T2 times compared to patients with preserved LVEF (60.8 ± 5.6 ms vs. 52.2 ± 2.8 ms; *p* = 0.02). Extracellular volume was significantly higher in patients with impaired LVEF (27.9 ± 2.1%) compared to those with preserved LVEF (22.7 ± 1.9%; *p* < 0.01). The authors suggested that septic cardiomyopathy with impaired LVEF reflects inflammatory cardiomyopathy However, there was no clear explanation for those with preserved ejection fraction who died, although the number of patients was very small (3 patients), making it difficult to draw any conclusions [135].

In a recent review, Lima et al. [137], reported that myocardial edema and inflammation are the key features of septic cardiomyopathy, with no evidence of focal fibrosis. In T2 sequences, homogeneous enhancement of the myocardium is seen, and after gadolinium application, no late myocardial thickening is observed.

### 4.8. Electrocardiography

Electrocardiographic findings are not diagnostic for septic cardiomyopathy. Sinus tachycardia and atrial fibrillation are the most common arrhythmias detected in electrocardiography in patients with sepsis [138].

### 4.9. Cardiac Biomarkers and Septic Cardiomyopathy

The most extensively studied cardiac serum biomarkers are troponin (I or T) and BNP (or NT-pro BNP) in the context of various heart diseases. Elevated cardiac troponin levels are associated with increased short-term mortality in critically ill, non-cardiac septic patients [139].

Troponin I (TnI) is often elevated in septic patients, likely due to multiple factors, probably non-ischemic mechanisms such as myocyte permeability, cell membrane leakage, apoptosis, refs. [26,140,141] and direct cellular toxicity induced by excessive catecholamine levels, renal dysfunction, and increased myocardial wall stress by volume overload [106]. Although troponins have been found to correlate with left ventricular systolic dysfunction, diastolic dysfunction, and RV dysfunction in a few studies using echocardiography in septic patients [72,141], results remain conflicting regarding whether troponin elevation or peak levels are directly associated with the diagnosis SCM [142,143,144].

Moreover, the association between troponin and hospital mortality is significantly reduced once confounding factors are controlled for elevated cardiac troponin levels are associated with increased short-term mortality in critically ill, non-cardiac septic patients [145]. However, once sepsis is diagnosed and other conditions known to elevate troponin, such as acute coronary syndrome, pulmonary embolism, or end-stage renal failure, are excluded [1], elevated cardiac troponin levels may help identify high-risk patients, who should undergo echocardiography.

Natriuretic peptides, including BNP, have been associated with prognostic value in patients with sepsis and potentially with SCM in some studies [19,21]. However, there is significant heterogeneity among the results regarding natriuretic peptide levels in sepsis, likely because concentrations above the reference range are common and are also linked to other conditions [21,146]. In a previous study [147], disease severity (as expressed by norepinephrine dosage, APACHE II, and SOFA scores), likely reflecting the intensity of inflammation, independently influenced BNP levels. Left and right ventricular ejection fractions were inversely related to BNP levels, but not independently, suggesting that septic cardiomyopathy is not the cause but coexists with elevated BNP in the context of severe inflammation. Furthermore, filling pressures did not correlate with BNP, indicating that BNP is not a marker of pulmonary edema or peripheral congestion [147].

Overall, increased cardiac biomarkers reflect the severity and prognosis of illness in patients with sepsis but are not specific for diagnosing SCM.

Other biomarkers have been studied in animal models. Fibroblast growth factor-21 (FGF-21) and growth differentiation factor-15 (GDF-15) have been identified as biomarkers reflecting the integrated mitochondrial response to stress [148]. Secretoneurin, a more recent biomarker, is elevated in states of myocardial dysfunction produced by neuroendocrine and myocardial tissues [149]. However, no studies have yet explored the use of secretoneurin in patients with SIMD.

## 5. Management of Sepsis and Sepsis-Induced Cardiomyopathy

The initial treatment of sepsis relies on early recognition and the application of etiological therapies, including targeted antibiotics and/or source control. Optimization of hemodynamic parameters—such as fluid replacement, administration of vasopressors, renal replacement therapies, and appropriate mechanical ventilation—is critical.

The Surviving Sepsis Campaign (SSC) Guidelines [1] recommend continuous hemodynamic monitoring using dynamic variables predictive of “fluid responsiveness” to guide fluid resuscitation [1,150]. Although there are user-friendly machines available in the ICU (e.g., PiCCO, FlowTrack) and algorithms for tracking pulse pressure variation (PPV) and stroke volume variation (SVV) in many advanced monitors, echocardiography plays a pivotal role.

**Figure 5:**
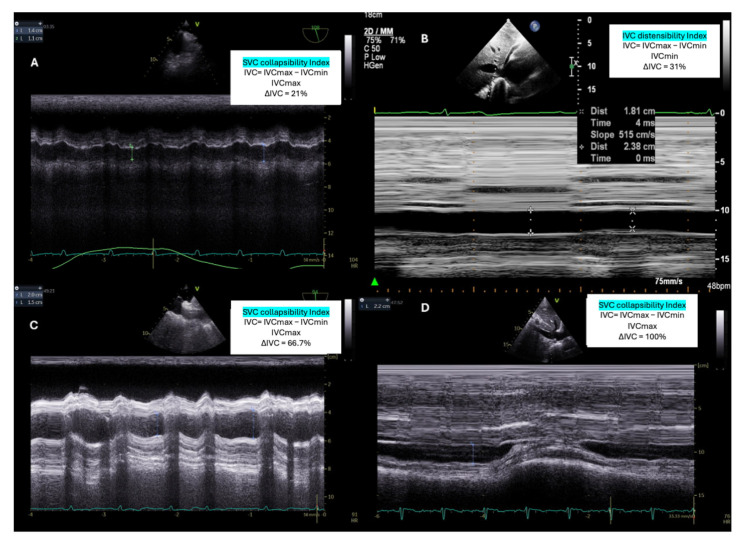
(**A**,**C**): Superior vena cava collapsibility index measured with transesophageal echocardiography. The reported threshold to identify fluid responsiveness varies in the literature between 18 and 36%. Patient (**A**) is probably not a fluid responder. On the contrary, patient (**C**) will respond to fluid administration. (**B**): IVC distensibility index in a fully sedated patient under controlled mechanical ventilation. The value of 31% indicates fluid responsiveness (**D**): IVC collapsibility index in a spontaneously breathing patient. The value of 100% indicates fluid responsiveness, although it should be considered with caution in a patient with rigorous respiratory efforts.

Echocardiography can identify the presence of SCM- albeit with recognized limitations that we have already mentioned-and predict fluid responsiveness using variables such as the distensibility index of the inferior vena cava (via transthoracic echocardiography) or the superior vena cava (via transesophageal echocardiography) [105]. It is also critical for identifying significant right ventricular (RV) failure, which may yield falsely positive results for “fluid responsiveness” based on PPV and SVV (Figure 5).

Following initial resuscitation, echocardiography assists in determining whether persistent hypovolemia or fluid responsiveness remains and helps establish when further fluid administration is unnecessary. For instance, echocardiographic indices such as inferior vena cava dynamics, SVV, and LV filling pressures (measured by E/e′) can optimize volume status and also to prevent fluid overload [105].

Although no specific treatment exists for probable SCM, initial fluid resuscitation with crystalloids is recommended. Intravenous fluid challenge of 20 mL/kg is used to increase preload and CO [1,5,150]. Volume replacement in the early stages is critical, but over-resuscitation in subsequent days can lead to complications such as pulmonary edema due to increased microcirculatory permeability and vasodilation [5,150].

The SSC strongly recommends norepinephrine as the first-line vasopressor to manage septic shock due to its predominant α-adrenergic effects, which improves vascular tone; arterial vasodilation is the main factor behind hemodynamic instability in septic patients [1,5]. Norepinephrine (NE)—an α- and β-agonist—has a stronger α-adrenergic profile compared with β-1, which results in an increase in afterload more than myocardial contractility [151]. While this is crucial for the treatment of distributive shock, the increased afterload may lead to a significant decrease in EF and CO, as has already been analyzed and will be further discussed below, resulting in the “unmasking” of previously latent SCM

However, norepinephrine’s side effects—including cardiotoxicity and peripheral ischemia—highlight the importance of cautious dosing [47,152]. Vasopressin (arginine) is suggested as a second-line vasopressor to supplement norepinephrine but is not advised yet as a first-line therapy.

Dopamine, though more β-1 selective, is no longer recommended due to its higher risk of arrhythmias and associated increased mortality [1].

### 5.1. Dobutamine

Dobutamine, a synthetic catecholamine with predominant β-1 effects [153], is recommended in cases of myocardial dysfunction presenting as high filling pressures and low CO, despite adequate fluid loading and norepinephrine use [1]. Change in myocardial adrenergic responsiveness has been reported in patients with septic shock [154]. Therefore, its efficacy in septic patients is variable, as adrenergic responsiveness is often impaired.

In an older study by Vieillard-Baron et al. [16], in a cohort of 67 septic shock patients, global hypokinesia was observed in 60% of the patients. Echocardiography was utilized to guide inotropic treatment. The administration of dobutamine combined with a reduced dose of norepinephrine (NE) improved LV hypokinesia, with hemodynamic improvement noted within 24 h. Conversely, in patients without hypokinetic states, the administration of NE alone induced global LV hypokinesia within 24–48 h. This phenomenon was explained by NE’s effect of increasing afterload, thereby unmasking potential myocardial failure. Based on these findings, the authors suggested that in cases of global hypokinesia, hemodynamic support should be adjusted by adding dobutamine to reduce NE dose [16].

In a recent review, the authors recommended cautious use of dobutamine in septic shock. Before making a final decision about its administration, the efficacy and tolerance of dobutamine should be evaluated over a short period under close monitoring for both its desired effects and potential side effects [155].

In conclusion, dobutamine may be considered in cases of persistent hypoperfusion despite adequate fluid resuscitation and NE administration. However, this approach is not strongly recommended and is supported by low-quality evidence [156]. In fact, only patients with global hypokinesia—probably due to SCM—should be tried with a low dose of dobutamine under close monitoring.

### 5.2. Levosimendan

Levosimendan, a calcium sensitizer, functions as both an inotrope (enhancing cardiac output without increasing myocardial oxygen demand) and a lusitrope, demonstrating anti-inflammatory, antioxidant, and anti-apoptotic properties. However, the Levosimendan for the Prevention of Acute Organ Dysfunction in Sepsis (LeoPARDS) randomized controlled trial (RCT) failed to show any benefit of levosimendan compared to standard care (including fluids, NE, and dobutamine) for improving organ dysfunction in patients with septic shock, regardless of LVEF or RVEF [157]. A subgroup analysis of patients with elevated cardiac biomarkers (troponin, NT-proBNP) confirmed this lack of efficacy [158].

The variability in cardiac function during sepsis or septic shock, ranging from hyperkinesia to normokinesia and hypokinesia, may partially explain the negative results of the trial. Inotropic therapy may not benefit patients with hyperkinesia or normokinesia [15], and cardiac biomarkers often rise in sepsis independently of cardiac dysfunction [147]. Therefore, the inclusion of a mixed population in the LeoPARDS study might have obscured levosimendan’s potential benefits for severe cardiac dysfunction [158].

Two meta-analyses conducted in 2017 and 2018 included randomized trials comparing levosimendan to other inotropes or standard therapy in patients with severe sepsis or septic shock. While levosimendan did not significantly reduce mortality, it effectively lowered serum lactate, improved cardiac contractility, and increased cardiac index and LVEF. However, norepinephrine requirements were not reduced, and levosimendan showed no superiority over dobutamine for mortality outcomes [159,160].

Another meta-analysis of seven studies, with the same objective as the previous ones, reported significantly reduced mortality with levosimendan compared to standard inotropic therapy (47% vs. 61%), along with lower blood lactate levels and higher cardiac index in the levosimendan group; no difference in mean arterial pressure and norepinephrine usage was noted [161].

However, all the above analyses lacked precise data regarding the severity of cardiac dysfunction and did not specifically compare levosimendan with dobutamine in patients with markedly impaired cardiac function.

In a recent observational study [162] on patients on septic shock with severe SCM (LVEF < 30% after fluid resuscitation), levosimendan treatment was compared with best available therapy. The levosimendan group, despite having more severely ill patients (higher APACHE II scores and lower LVEF at baseline), showed a significant improvement in LVEF after seven days compared to no improvement in the control group. Additionally, lactate levels decreased significantly within the first 24 h. These findings suggest levosimendan may be effective for extremely severe SCM, warranting consideration of ECMO if available [162].

Similarly, a study by Sun et al. [163] compared levosimendan and dobutamine in patients with SCM (LVEF ≤ 35%). At 24 h, the levosimendan group demonstrated higher cardiac index, LVEF, stroke volume index, and fluid volume, with lower norepinephrine requirements compared to the dobutamine group. On the third day, cardiac troponin I levels were lower in the levosimendan group.

Although neither study showed significant differences in mortality between levosimendan and dobutamine, levosimendan was more effective in improving cardiac function and reducing norepinephrine requirements. It should be noted, however, that these studies included only a small number of cases with extremely severe SCM. These findings highlight the potential efficacy of levosimendan for severe SCM, though further studies are required to confirm its impact on clinical outcomes.

### 5.3. Beta-Blockers and Ivabradine in Septic Shock

The adrenergic system plays a critical role in sepsis, often provoking tachycardia and tachyarrhythmias, which can exacerbate left ventricular (LV) systolic and diastolic dysfunctions. β-adrenergic modulation may be considered as a therapeutic intervention in these cases. Short-acting beta-blockers, such as esmolol [164,165] and landiolol [166,167], are preferred due to their effectiveness in reducing heart rate. However, their use carries the risk of worsening hemodynamic instability, which necessitates cautious administration. These beta-blockers have been evaluated for their impact on tachyarrhythmias with the objective of improving outcomes in patients with septic shock [50,168].

A meta-analysis of seven randomized controlled trials reported that esmolol and landiolol use in patients with sepsis and persistent tachycardia was associated with reduced 28-day mortality [50]. Conversely, a very recent updated meta-analysis from the same group, including subsequent multicenter RCTs reporting conflicting findings, the use of esmolol or landiolol did not reduce mortality in patients with sepsis with persistent tachycardia. However, results were not robust and outcomes differed between single-center and multicenter RCTs [169]. Similarly, a recent study evaluating these ultra-short-acting β-blockers in septic shock did not demonstrate a favorable effect on either SOFA scores or 28-day mortality [170]. Importantly, none of these studies specifically evaluated their effects on probable SCM. Therefore, further studies regarding ultra-short-acting β-blockers with advanced cardiac monitoring and serial echocardiography are warranted.

In the study by Morelli et al. [165], esmolol improved hemodynamics and significantly increased survival in patients with septic shock, yet heart failure patients were excluded. Similarly, in Kakihana et al.’s study [166], where mean LVEF was 55% ± 15%, cardiogenic shock was an exclusion criterion, although patients with relatively low LVEF were included. The study primarily aimed to investigate the safety and efficacy of landiolol in treating sepsis-related tachyarrhythmias (atrial fibrillation, atrial flutter, or sinus tachycardia), but no separate subgroup analysis for patients with low LVEF was performed. Adverse events were reported in 64% of patients treated with landiolol, including serious adverse events in 12%, such as hypotension, cardiac arrest, and reduced ejection fraction. The authors concluded that while landiolol was generally well-tolerated, its use requires careful monitoring of blood pressure and heart rate due to the risk of hypotension in sepsis and septic shock patients.

In another small study involving nine patients in the early hyperkinetic phase of septic shock, esmolol was associated with exacerbated hypotension and a decreased cardiac index [171]. Thus, beta-blockers should be administered with extreme caution, particularly in cases with systolic dysfunction, as this may contraindicate their use in sepsis and septic shock patients [172].

Morelli et al. [173], introduced the systolic-dicrotic notch pressure difference as a method to identify tachycardic patients at risk of decompensation when heart rate reduction is attempted. A smaller difference was associated with compensatory tachycardia due to reduced LV contractility, increasing the risk of decompensation. This difference, measured by the arterial dP/dt max (derived from the radial arterial pressure waveform contour), was proposed as a surrogate marker for ventriculoarterial coupling [173].

Ivabradine, a selective If channel inhibitor in the sinoatrial node, offers another therapeutic option. Unlike beta-blockers, it lowers heart rate without negatively affecting myocardial contractility. In a small case series of three patients, ivabradine improved heart rate, stroke volume index, end-diastolic volume index, and SvO2 while reducing lactate levels and norepinephrine requirements [174]. However, a larger trial, which included mixed subgroups of cardiac and septic shock patients, suggested a trend toward worsened mortality with ivabradine use [175].

Finally, an animal study comparing ivabradine and atenolol to placebo in a sepsis model revealed that while both blunted tachycardia, atenolol reduced cardiac output, systolic blood pressure, and LV systolic function compared to placebo. Neither intervention demonstrated survival benefits compared to placebo [176]. Thus, while ivabradine appears to have fewer hemodynamic drawbacks than beta-blockers, further evidence is needed to establish its efficacy and safety in septic shock and SCM.

### 5.4. Extracorporeal Membrane Oxygenation (ECMO)

Early venoarterial extracorporeal membrane oxygenation (VA-ECMO) may be a viable option for certain patients with refractory sepsis-induced cardiogenic shock, especially when conventional therapies fail to stabilize hemodynamics [177,178,179].

In an older study, VA-ECMO was implemented in 14 patients with refractory septic shock, characterized by severely reduced left ventricular ejection fraction (LVEF < 25%), low cardiac index (CI < 2.2 L/min/m^2^), persistent hypotension, extensive skin mottling, or elevated lactate levels despite high-dose catecholamines. The median ECMO duration was brief (5.5 days [2–12 days]), and when VA-ECMO was discontinued, the median LVEF had significantly improved to 60% (40–70%), suggesting the reversibility of SCM. Based on these findings, the authors hypothesized that VA-ECMO provides critical time to reverse SCM by restoring adequate perfusion to vital organs [178].

In a retrospective, multicenter controlled trial involving 82 patients with sepsis-induced cardiogenic shock refractory to conventional treatments (defined by LVEF < 35% and severe hemodynamic compromise, including persistent lactic acidosis despite high doses of catecholamines and aggressive fluid therapy), the use of VA-ECMO significantly improved 90-day survival compared to controls not receiving ECMO (mortality: 60% vs. 25%, *p* = 0.0029) [66]. VA-ECMO allowed rapid reduction in vasopressor requirements, restoration of vital organ perfusion, and improvement in cardiac function. Survivors were successfully weaned off ECMO after a median of six days [177].

A meta-analysis of 14 observational studies, encompassing 468 patients, reported an overall pooled survival rate of 36.4%. Notably, survival was significantly higher among patients with LVEF < 20% than in those with LVEF > 35%. Regional differences were observed, with survival rates in Asia (19.5%) being considerably lower compared to Europe (61.0%) and North America (45.5%). These variations emphasize the need for cautious interpretation of the data [179].

Given these findings, early identification of patients with SCM characterized by extremely severe sepsis-induced cardiac dysfunction and refractory septic shock—which might account for up to 10% of all septic shock cases—is critical. Prompt initiation of VA-ECMO in this subgroup could play a pivotal role in improving outcomes [177].

## 6. Phenotypes: Can They Reduce Heterogeneity in Sepsis?

Sepsis is a heterogeneous syndrome. Identifying distinct clinical phenotypes may enable more precise therapies and improve patient care.

Several research groups have attempted to define subgroups using clinical data such as demographics, organ dysfunction, vital signs, and laboratory values [180,181,182,183]. However, only a few studies have introduced echocardiography with the aim of revealing clusters that may require different monitoring and management.

Geri et al. [184], conducted a study utilizing transesophageal echocardiography during the initial phase of septic shock to identify five distinct cardiovascular phenotypes, or clusters, based on clinical and echocardiographic parameters, ICU mortality was evaluated. Cluster 1 comprised 16.9% of patients who were well-resuscitated and exhibited no evidence of left ventricular systolic dysfunction, right ventricular failure, or fluid responsiveness, ICU mortality of 21.3%. Cluster 2, representing 17.7% of the cohort, included patients with left ventricular systolic dysfunction, who had a significantly higher ICU mortality of 50%. Cluster 3 accounted for 23.3% of patients and was characterized by a hyperkinetic profile, with lower mortality rates 23.8% in the ICU. Cluster 4 included 22.5% of patients who exhibited right ventricular failure, ICU mortality of 42%. Finally, cluster 5 represented 19.4% of patients with persistent hypovolemia, showing mortality rates of 38.6% in the ICU. The study revealed significant differences in mortality outcomes across the clusters, with clusters characterized by left ventricular systolic dysfunction and right ventricular failure exhibiting the highest mortality rates.

As already has been mentioned Dugar et al., ref. [70] in a retrospective cohort study using transthoracic echocardiography divided septic patients into five groups based on LVEF (LVEF < 25%, 25% ≤ LVEF < 40%, 40% ≤ LVEF < 55%, 55% ≤ LVEF < 70%, and LVEF ≥ 70%). The association of LVEF to in-hospital mortality in sepsis and septic shock was U-shaped. Both severe LV systolic dysfunction (LVEF < 25%) and hyperdynamic LVEF (LVEF ≥ 70%) were associated independently with significantly higher in-hospital mortality. Recently, Sato et al. from the same group of the previous authors, investigated the prevalence and association of hyperdynamic LV systolic function- defined using LVEF of 70% as cutoff- with mortality in patients with sepsis in a systematic review and meta-analysis. The prevalence of hyperdynamic LV systolic function was associated with significantly higher short-term mortality as compared to normal LV systolic function.

No difference was found in E/e’ between normal and hyperdynamic LV systolic function, while higher values of heart rate and LVEDD were detected in patients with hyperdynamic LV systolic function [185]. However, in these patients with hyperdynamic LV systolic function patients, probably EF may be the mirror of a hyperadrenergic state driven by excessive endogenous and exogenous catecholamine stimulation due to the severity of the shock. Of course, SCM may exist in these patients too, as indicated by the increased LVDD without increased LVFP as indicated by E/e’ between normal and hyperdynamic patients. Newer methods are needed to discriminate this cluster (if so) of patients.

These findings underscore the heterogeneity of cardiovascular phenotypes in septic shock and highlight the importance of tailored interventions informed by echocardiographic monitoring to improve patient outcomes.

Recently, Tsolaki et al. retrospectively evaluated cardiac function in 62 mechanically ventilated patients with sepsis or septic shock caused by multidrug-resistant (MDR) or non-MDR pathogens. Two distinct SCM phenotypes emerged based on antimicrobial resistance. LV systolic dysfunction was more severe in the non-MDR-SCM group, with lower LVEF, LV systolic dysfunction and LV strain, while the MDR-SCM group exhibited more pronounced RV dysfunction, including greater RV dilatation, lower RVFAC, reduced systolic tissue Doppler velocity and more impaired RV strain. no significant difference in mortality was found [186].

Recent research into gene expression in sepsis has highlighted differential activation of pathways related to immunity and metabolism, and endotoxin tolerance, which may correspond to distinct phenotypic clusters [187,188,189,190]. These findings suggest that the observed clinical and echocardiographic phenotypes may reflect underlying pathophysiological variations that could guide more precise therapeutic interventions.

Understanding the differences in cardiac responses to sepsis is critical for developing more personalized strategies for hemodynamic resuscitation. Tailored approaches may improve outcomes by addressing the distinct underlying mechanisms in each phenotype. For example, fluid restriction might benefit patients with LV systolic or RV dysfunction to avoid exacerbating cardiac overload, while inotropes may be effective specifically in cases of systolic dysfunction. Arginine-vasopressin could be prioritized over norepinephrine in RV dysfunction because it does not induce vasoconstriction in the pulmonary vasculature. In hyperdynamic states or cases with LV outflow tract obstruction, beta-blockers could be considered, provided there is adequate fluid resuscitation. Antimicrobial therapy should also be adjusted to target MDR pathogens more effectively, especially given the differing impacts of MDR and non-MDR infections on cardiac function.

Despite these promising developments, the therapeutic strategies in sepsis and septic shock remain largely theoretical. The various attempts to classify patients into clusters based on clinical, echocardiographic, or molecular criteria underscore the need for further research to validate these subgroupings. By integrating these findings, future approaches may move toward personalized treatments that consider the specific characteristics and needs of individual patients.

## 7. Artificial Intelligence to Help Diagnose and Predict SCM in Clinical Practice

Artificial Intelligence (AI) technologies hold significant potential in diagnosing and predicting clinical settings where early detection and accurate prognosis are crucial for improving patient outcomes.

In clinical practice, multiple sources of patient data (such as lab results, biomarkers, imaging such as echocardiograms, genes, clinical notes, and vital signs) are often scattered across different systems. AI technologies, with machine learning models trained on large datasets, particularly those employing natural language processing (NLP) and data integration algorithms, can pull together diverse datasets, offering a holistic view of a patient’s health status.

Until now, AI has not extensively used to SCM. Chen et al., intended to identify biomarkers for SCM Based on Bioinformatics Analyses. Differentially expressed genes (DEGs) between SC and control groups were identified, followed with functional enrichment analyses. The upregulated DEGs were significantly enriched tumor necrosis factor signaling pathway, Jak-signal transducer and activator of transcription signaling pathway, hypoxia-inducible transcription factor-1 signaling pathway, chemokine signaling pathway, and cytokine-cytokine receptor interaction, concluding that the identified DEGs and pathways may be implicated in the progression of human SC, which may lead to a better understanding of SC pathogenesis [191].

Li et al., employed bioinformatics analysis and drug discovery approaches to identify the regulatory molecules, distinct functions, and underlying interactions of mitochondrial metabolism and immune microenvironment, along with potential interventional strategies in SCM. GSE79962, GSE171546, and GSE167363 datasets were obtained from the Gene Expression Omnibus (GEO) database. DEGs, six hub mitochondria-related DEGs, and module genes were identified, concluding that in the study presented a comprehensive mitochondrial metabolism and immune infiltration landscape in SCM, providing a potential novel direction for the pathogenesis and medical intervention of SCM [192]

Finally, Li et al., used integrated multi-omics analysis to explore the clinical and genetic roles of efferocytosis in SCM. They identified six module genes (ATP11C, CD36, CEBPB, MAPK3, MAPKAPK2, PECAM1) strongly associated with SCM. They identified a novel efferocytosis-related SCM subtype and diagnostic biomarkers, offering new insights for clinical diagnosis and therapy. Moreover, experimental validation supported computational results [193].

In summary, AI’s potential to diagnose and predict septic cardiomyopathy may lie in its ability to analyze complex, multidimensional data to detect early signs of cardiac dysfunction, predict disease progression, and guide personalized treatment plans. As AI tools become more refined and widely adopted, they have the potential to significantly improve clinical decision-making, enhance patient care, and ultimately may reduce the burden of SCM in critically ill patients.

## 8. Conclusions

Septic cardiomyopathy is a complex and poorly defined condition with significant implications for the management and outcomes of patients with sepsis and septic shock. The absence of standardized diagnostic criteria, coupled with conflicting data on diagnostic tools such as echocardiography and biomarkers, limits the accurate identification and management of SCM. Current therapies remain supportive, focusing on hemodynamic optimization and infection control, while promising approaches like levosimendan and VA-ECMO require further validation.

Future research should prioritize the establishment of clear diagnostic frameworks, integrating echocardiographic parameters, biomarkers, and clinical clustering to enhance understanding of underlying pathophysiology. Tailored treatment strategies based on these findings have the potential to significantly improve clinical outcomes in SCM, particularly for patients with severe septic shock. As our understanding of SCM evolves, these advancements will pave the way for more effective and individualized management approaches.
